# Poster Session II - A256 THE IMPACT OF FISTULAS IN PATIENTS WITH ILEAL POUCH ANAL ANASTOMOSIS: A COMPARATIVE COHORT STUDY

**DOI:** 10.1093/jcag/gwaf042.256

**Published:** 2026-02-13

**Authors:** N Sayed, D Unninayar, R Abad-Fujiyoshi, M Narbonne, R Ghasemi, S Murthy, J McCurdy

**Affiliations:** Ottawa Hospital, Ottawa, ON, Canada; Ottawa Hospital, Ottawa, ON, Canada; Ottawa Hospital, Ottawa, ON, Canada; Ottawa Hospital, Ottawa, ON, Canada; Ottawa Hospital, Ottawa, ON, Canada; Ottawa Hospital, Ottawa, ON, Canada; Ottawa Hospital, Ottawa, ON, Canada

## Abstract

**Background:**

Ileal pouch-anal anastomosis (IPAA) is the standard surgical approach for ulcerative colitis. Complications associated with IPAA, including pouchitis, strictures, and fistulas pose major risks for morbidity, diversion, and IPAA failure. However, the impact of fistulas on IPAA outcomes are poorly understood.

**Aims:**

To determine the cumulative incidence of IPAA associated fistulas (IAFs), and to determine if they are associated with adverse IPAA outcomes.

**Methods:**

We performed a retrospective cohort study of adults with inflammatory bowel disease (IBD) who underwent IPAA and were followed at The Ottawa Hospital (2005-2024). Fistulas were classified as perianal, rectovaginal, or pouch-body, and as early (<6 months) or late (>6 months) post-ileostomy closure. Kaplan-Meier methods estimated cumulative incidence of fistula formation, fecal diversion, and pouch excision. Cox proportional hazards models adjusted for age, sex, luminal Crohn’s-like phenotype, and rectal stricture to evaluate the association between IAFs and pouch failure, a composite of fecal diversion or pouch excision. Fistula remission was defined clinically as absence of drainage without diversion.

**Results:**

We identified 332 patients with IPAA: 48.8% female, mean age 33 ± 13 years. The cumulative risk of developing a IAF was 3.1%, 8.4%, 13.8%, and 29.8% at 1, 5, 10, and 20 years, respectively (Figure 1). The types of fistulas included perianal (55.8%), rectovaginal (37.7%), and pouch-body (6.5%). After a mean follow-up of 15.5 ± 9.9 years, PAFs were strongly associated with pouch failure (aHR, 7.1; 95% CI 3.3-15.5) including fecal diversion (aHR, 5.3; 95% CI 2.2-12.5) and pouch excision (aHR, 9.9; 95% CI 3.2-31.0). Following fistula diagnosis, 70% received an advanced therapy, most often anti-TNF therapy (87%). Fistula remission without fecal diversion occurred in 37.5% at 3 months, 39.6% at 6 months, and 41.7% at 12 months after first-line advanced therapy; 27.1% remained in remission at final follow-up.

**Conclusions:**

In this single center study, we found that 14% of patients developed an IAF within 10 years and that fistulas were significantly associated with IPAA failure. These findings highlight the need for early detection, multidisciplinary care, and development of targeted therapies for fistulizing pouch disease.

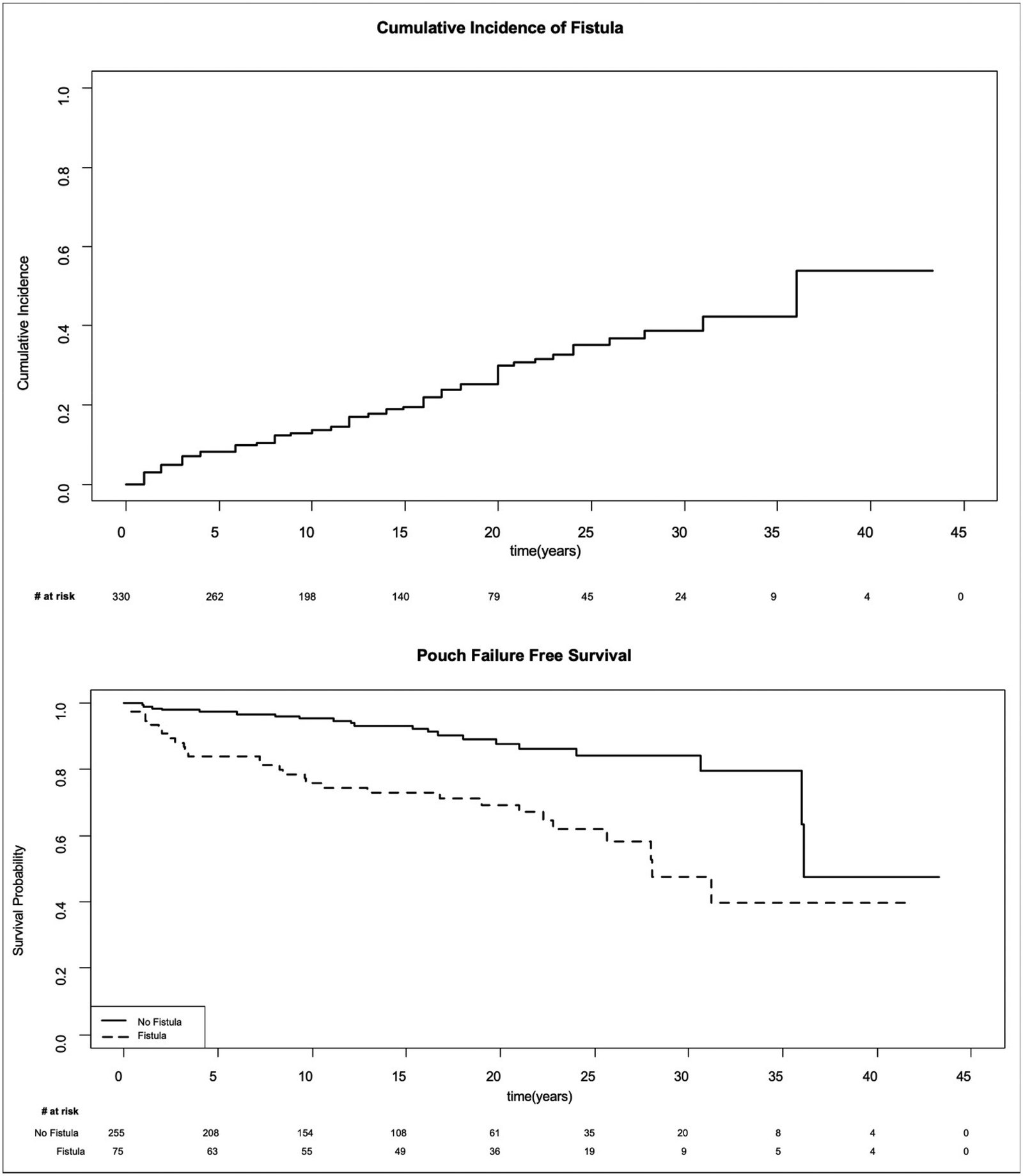

**Funding Agencies:**

None

